# Investigating Wearable Sensor Placement for Heat Risk Monitoring of Firefighters: A Skin-Ear Temperature Study

**DOI:** 10.3390/s26154837

**Published:** 2026-07-31

**Authors:** W. D. Ruwandi Fernando, Marianella Chamorro-Koc, Ajay K. Pandey, Daniel Cook

**Affiliations:** 1 School of Design, Faculty of Creative Industries, Education and Social Justice, Queensland University of Technology, Brisbane, QLD 4000, Australia; m.chamorro@qut.edu.au (M.C.-K.); d20.cook@qut.edu.au (D.C.); 2 School of Electrical Engineering and Robotics and Faculty of Engineering, Queensland University of Technology, Brisbane, QLD 4000, Australia; a2.pandey@qut.edu.au

**Keywords:** core body temperature, skin temperature, sensor placement, wearable, heat

## Abstract

**Highlights:**

**What are the main findings?**

- Afternoon conditions showed lower variability between ST and CBT, suggesting time-of-day influences sensor placement reliability.
- Non-calm conditions showed lower variability between ST and CBT compared to calm states, indicating activity level affects sensor performance.
- Greater variability in ST and CBT for females highlights the need to consider sex in sensor placement.

**What is the implication of the main finding?**

- Elbow sites outperformed wrist locations, suggesting a clearer hierarchy for on-body sensor placement in heat risk monitoring.

**Abstract:**

In firefighting, heat stress is a significant physiological hazard that leads to an increased risk of heat-related illness. Heat-risk monitoring of core body temperature (CBT) can help identify early signs of heat strain and can be accomplished with wearable sensing technologies (WST). This study employs non-invasive temperature sensing to examine the relationship between tympanic temperature and skin temperature (ST) and across multiple on-body locations under different experimental conditions. This study was conducted with 10 healthy adults, male and female, under calm and non-calm conditions, at different times of the day, and across multiple sessions, representing moderate physiological strain similar to firefighting training activities. Tympanic temperature was used as a practical proxy for CBT and compared with ST measured at the forehead, wrist, and elbow. Bland–Altman analysis, together with the variance of differences between CBT and ST, was used to identify the most reliable on-body sensor location. Elbow sites showed lower variability for CBT than wrist sites, suggesting greater stability and suitability for wearable deployment in the context of firefighters’ gear in situations where forehead placement is considered impractical. Results show that variability is affected by activity state, time of day, sex, and anatomical location. Findings consist of preliminary evidence informing the most suitable placement of a wearable sensor for heat risk monitoring.

## 1. Introduction

Firefighting is a safety-critical occupation performed under conditions that place substantial physiological demands on the body. Exposure to extreme heat, intense physical exertion, and reduced visibility can rapidly elevate thermal strain and increase the risk of heat-related illness, cognitive impairment, and cardiovascular stress [1,2,3]. These risks may be more pronounced among firefighter trainees, whose limited operational experience can reduce their ability to recognize and manage heat stress effectively [4,5]. The challenge is further compounded by the use of encapsulating personal protective equipment (PPE), which restricts heat dissipation and contributes to rising core body temperature during fire operations. This suggests that continuous monitoring of heat strain is therefore an important component of firefighter safety and training [4]. However, existing wearable sensing approaches remain difficult to implement in operational contexts. Direct methods for measuring core body temperature (CBT) such as rectal temperature measurement and telemetry pills, although often accurate, are frequently intrusive, uncomfortable, or impractical for routine field deployment [6,7]. These limitations suggest the need for technology sensing approaches that can provide meaningful physiological information while remaining compatible with the practical demands of firefighting environments. Therefore, this article presents an exploratory study investigating non-invasive skin and ear temperature sensing to examine sensor placement for wearable technology.

A range of techniques have been proposed to estimate or approximate core body temperature across domains such as medicine, sports science, mining, surgery, and occupational safety [7,8,9,10,11,12,13,14,15]. These techniques include direct measurement techniques such as esophageal and rectal thermometry, ingestible telemetry pills, and tympanic temperature sensors, which provide varying levels of accuracy but differ significantly in usability and deployment feasibility. In firefighting research, rectal thermometry and ingestible sensors are commonly treated as reference methods because of their relative accuracy in approximating CBT [7,12]. Nevertheless, their invasive nature and operational limitations constrain their suitability for continuous wearable monitoring in real-world settings. Consequently, recent work has increasingly investigated indirect estimation approaches that infer thermal strain from physiological signals such as heart rate and skin temperature (ST) [15,16,17]. While these methods present opportunities for less intrusive wearable systems, their performance remains sensitive to environmental conditions, sensor placement, movement, and inter-individual variability. Such challenges become particularly significant in firefighting contexts, where high thermal loads, physical activity, and PPE introduce additional complexity and uncertainty into physiological measurements.

These limitations point to a broader challenge in wearable physiological sensing: ensuring that measurements remain both reliable and practically usable under realistic operational conditions. A report published by NFPA (USA) indicates several health risks in firefighting conditions, where overexertion is identified as the main cause of casualties; however, these reports do not yet include the use of technology as a resource to manage safety risks [4]. In safety-critical environments, wearable systems must function without interfering with movement, task execution, or protective equipment, while still providing physiologically meaningful data. Heat-strain monitoring can therefore be understood not only as a sensing problem, but also as a wearable systems design problem in which performance depends on how sensing technologies are physically integrated with the body and operational context. Wearable sensing technologies (WST) are body-worn systems intended to continuously acquire physiological information while remaining acceptable, unobtrusive, and usable in practice [6,18]. As a result, their design requirements extend beyond sensing accuracy to include wearability, usability, comfort, reliability, and contextual suitability. Earlier studies on wearability primarily focused on physical integration with the body, including fit, comfort, and minimizing disruption to movement [19]. More recent perspectives further emphasize long-term comfort, user acceptance, and the lived experience of using wearable devices within real operational environments [18,20]. A sensing system may achieve strong technical performance in controlled settings yet still prove unsuitable in practice if it is uncomfortable, intrusive, or incompatible with operational routines [15,21]. Therefore, although the chest and abdomen may better approximate CBT under controlled conditions, these sites were not considered due to the practical challenges of integrating sensors beneath multiple layers of firefighter PPE and the self-contained breathing apparatus (SCBA) harness, which can affect sensor placement, comfort, and accessibility.

Within firefighting environments, three interrelated design considerations are particularly relevant for wearable heat-monitoring systems: *comfort*, *sensor placement*, and *reliability*. Comfort relates to the physical integration of the device with the body, including size, weight, attachment, and freedom of movement. Because firefighters already operate while carrying substantial PPE loads, wearable systems must minimize additional physical burden and remain compatible with existing protective equipment. Placement concerns the selection of body locations that provide both physiologically meaningful signals and practical feasibility for wearable integration. Sensor location influences signal quality, stability, susceptibility to motion artefacts, attachment reliability, and compatibility with PPE. Reliability encompasses both measurement consistency and the extent to which sensed data provide interpretable and useful information about heat strain. Together, these considerations illustrate the interconnected nature of sensing performance and wearable design in operational contexts.

Against this background, the present study investigates ST and tympanic (ear) temperature sensing as relatively accessible and minimally intrusive approaches for heat monitoring in firefighting-related conditions. Tympanic temperature is considered here as a practical field-relevant proxy for CBT, while ST is measured at multiple candidate body locations including the forehead, wrist, and elbow [22,23,24,25,26]. These locations were selected on the basis of physiological considerations, with upper limb sites such as the wrist and elbow offering higher thermal responsiveness and thereby providing valuable insights into the body’s overall thermal state [25,27]. Particular attention is given to factors such as activity level, time of day, and individual variability, including gender-related differences, all of which may influence signal behavior and interpretation. Understanding these variations is important for wearable system design, as sensing approaches that assume stable relationships between peripheral and internal temperature measurements may not generalize effectively in operational settings.

Accordingly, this exploratory study focuses on the sensor placement of a non-invasive heat risk monitoring wearable sensor. By statistically assessing the agreement between ST measurements and tympanic temperature across multiple body locations, the study investigates whether certain skin sites demonstrate closer correspondence with internal thermal status than others under the tested conditions. The intention is not to position ST as a direct replacement for CBT measurement, but rather to explore whether some body locations may offer more informative and practically feasible signals for wearable heat monitoring. In doing so, the study contributes empirical evidence to support sensor placement decisions and to evaluate the potential usefulness of these measurements for heat-risk monitoring in wearable applications. Drawing on perspectives from physiology, sensor engineering, and wearable systems design, this work adopts an interdisciplinary exploratory approach intended to contribute to the future development of wearable heat-monitoring technologies for firefighter training and safety applications [18,22,23,24,25].

The remainder of this paper is structured as follows. Section 2 describes the study methodology, Section 3 presents the experimental results, Section 4 discusses the findings, and Section 5 concludes the paper and outlines limitations and directions for future work.

## 2. Materials and Methods

### 2.1. Ethical Statement

The study protocol was approved by the University Human Research Ethics Committee (UHREC) at Queensland University of Technology (Approval number is 10265).

### 2.2. Study Design

This study aims to explore non-invasive temperature sensing based on infrared by examining correlations between tympanic temperature, a medically accepted CBT proxy [22,23,24,26], and STs at selected body sites (mid-forehead, wrist, and elbow). These sites were selected based on their thermal responsiveness, relevance in existing wearable technologies, ease of access, and support from previous literature for providing meaningful insights into the body’s thermal state [25,27].

### 2.3. Participant Recruitment

A total of 10 adult volunteers were recruited for this study. Participants were healthy individuals aged between 26 and 45 years, with 50% female representation. In Australia, the usual age range for participants is 18–65. Firefighters typically begin their careers in their mid-20s, and only Queensland has a mandatory retirement age for firefighters. Therefore, the age range of 26–45 was chosen as representative of the working age of firefighters in Australia. This decision was informed through consultation with Queensland Fire and Emergency Services, as this data was not publicly available. Due to the study requiring consistent environmental temperature and humidity levels, and the need to collect the participants’ CBT and ST four times a day, participant recruitment focused on healthy adults within the same workspace. This presented a limitation to this study in that participants were not firefighters and were not wearing firefighting gear. Participants wore clothing that allowed easy access to their arms to facilitate temperature measurements in their workspace.

### 2.4. Data Collection

**Figure 1:**
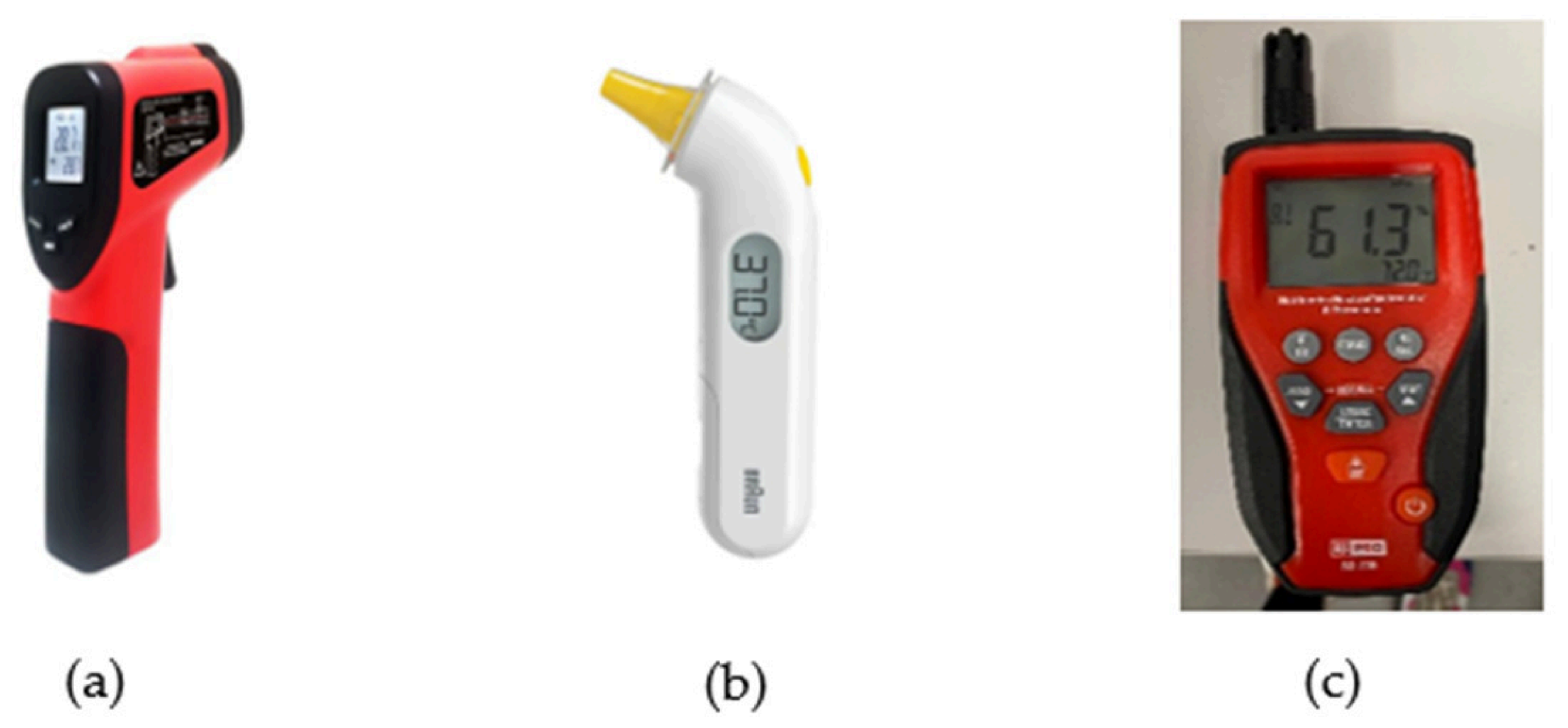
Devices used: (**a**) infrared thermometer, (**b**) clinical tympanic thermometer, (**c**) moisture meter.

Data collection was conducted at an Engineering Laboratory at the university where the authors work. STs and CBTs were measured using non-invasive devices. ST at selected sites was measured using a non-contact infrared thermometer (Matador Surface Infrared Thermometer) (Matador, Prestons, NSW, Australia) (Figure 1a). CBT was measured using a clinical tympanic thermometer (Braun ThermoScan^®^ 3) (IRT3030; Famidoc Technology Co., Ltd., Dongguan, China) (Figure 1b). Both devices are commonly used in domestic healthcare settings and pose no risk to participants [22]. Ambient temperature and relative humidity levels within the testing environment were recorded four times per day using a moisture meter (RS239) (Model RS239; RS Components Ltd., Shanghai, China) (Figure 1c).

**Figure 2:**
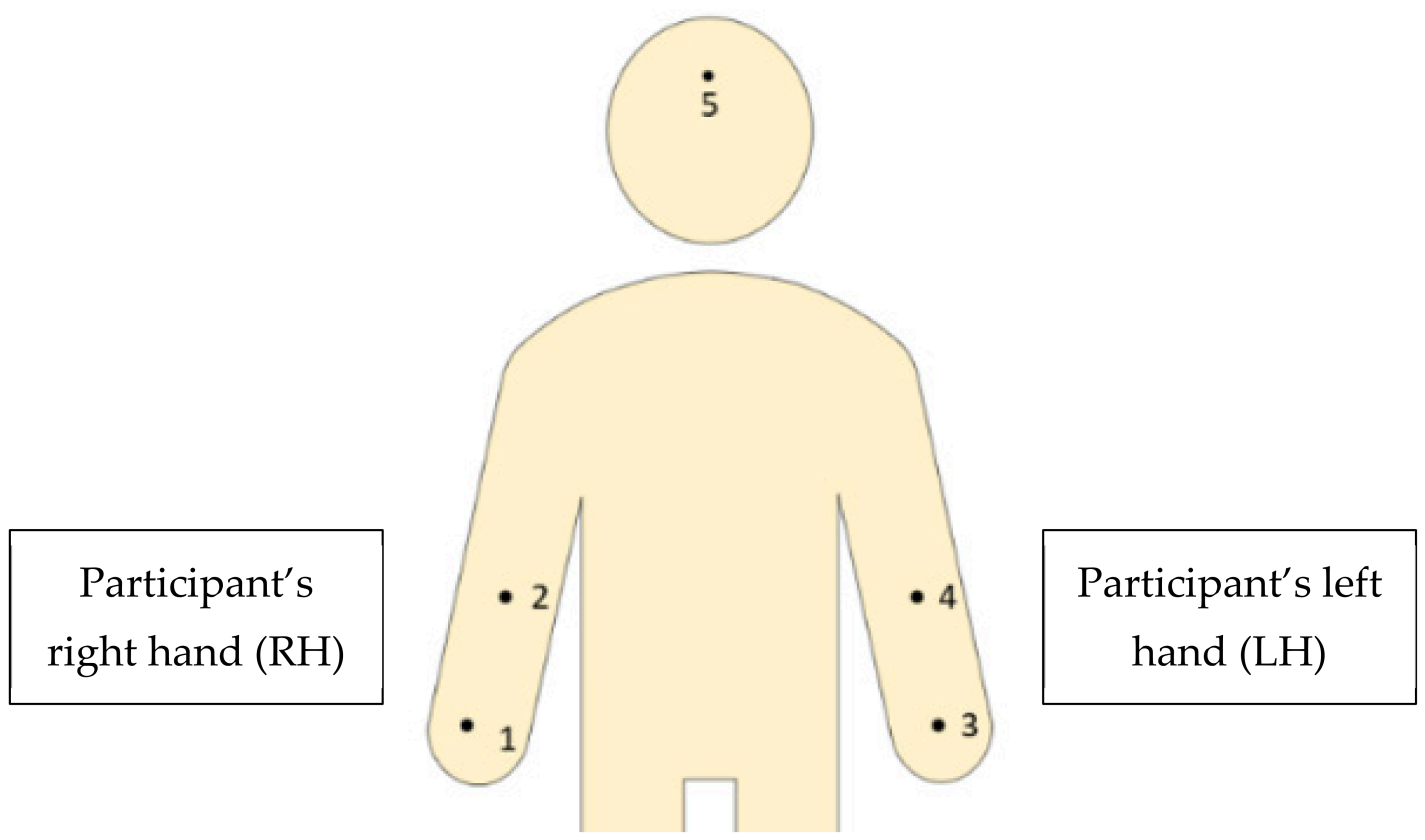
Data points for measurements (front view).

Temperature measurements were collected from multiple body locations. ST was recorded at the wrist and elbow of both arms (four sites in total) and at the midpoint of the forehead (one site) (Figure 2). Tympanic temperature was measured in both ears. In total, seven temperature measurements were collected during each measurement session. Measurements were taken four times per day for three consecutive days. Thermometer probe covers were disposed of between measurements.

The planned recruitment of 10 participants is based on methodological precedent. Similar studies have shown that reliable correlational analysis can be achieved with repeated measures across a small cohort [10,28,29]. Temperature measurements were conducted from morning to evening based on participant availability, with readings taken at approximately two-hour intervals, resulting in four measurements per day. Each measurement session required approximately 2–3 min per participant.

Two physiological states were considered during data collection: calm and non-calm. The calm state involved measurements taken during normal resting conditions without additional physical activity. The non-calm state involved a short period of controlled physical exertion prior to measurement, where participants performed a five-minute stair walk to induce a moderate increase in physiological demand. Stair climbing is a functional exercise task in occupational and physiological research because it elicits increased cardiovascular and metabolic responses while being simple, repeatable, and practical to implement. In firefighting contexts, stair climbing represents an important component of operational activities, including high-rise access, equipment transport, and emergency response; however, it represents only one aspect of the complex physical demands experienced during firefighting. Therefore, the stair-walking protocol was selected as a controlled exercise challenge to represent a component of firefighter-related physical workload rather than a complete simulation of occupational firefighting conditions [30,31]. Calm and non-calm states alternated across measurement sessions throughout the day. Measurements were conducted individually for each participant over three consecutive days. The experiment was carried out in a room located in close proximity to the participant.

### 2.5. Data Analysis

The analysis aims to investigate the relationships between tympanic temperature, used as a proxy for CBT, and STs measured at selected body locations, including the mid-forehead, wrist, and elbow. Understanding these relationships is important for identifying skin measurement sites that may provide a reliable indication of the body’s thermal state using non-invasive infrared sensing. Temperature measurements were collected from multiple participants across several time points over five consecutive days under both calm and non-calm conditions. The dataset therefore includes variations in physiological state and environmental conditions, allowing for a more representative assessment of temperature responses.

According to the study design, there are eight scenarios, as follows:

A—Morning-Female-Calm;
B—Morning-Female-Non-Calm;
C—Afternoon-Female-Calm;
D—Afternoon-Female-Non-Calm;
E—Morning-Male-Calm;
F—Morning-Male-Non-Calm;
G—Afternoon-Male-Calm;
H—Afternoon-Male-Non-Calm.

#### Statistical Tools Used for Analysis

The analysis focuses on identifying patterns and agreement between tympanic and skin temperature measurements across the different body sites. Descriptive statistics and analysis were used to evaluate the strength of the relationships between the variables and to determine which measurement locations may best reflect changes in core body temperature. Variance analysis of ST and CBT was initially performed for each scenario and measurement site to assess the consistency of the dataset (Table 1). To determine the extent of agreement between ST and CBT and to identify the skin site that most closely reflects CBT, two further analyses were performed for each scenario and site: (1) calculation of the variance of the CBT–ST difference (Table 2), and (2) Bland–Altman analysis to determine bias and limits of agreement (LoA) (Table 3). These analyses provided a basis for evaluating both the variability of the measurements and the suitability of each skin site as a proxy for CBT [32]. The results of this analysis are presented in the following subsections.

## 3. Results

Overall, the results suggest that the relationship between ST and CBT, represented in this study by tympanic temperature, varies depending on contextual conditions and measurement-related factors. In particular, differences were observed with respect to time of day, activity condition, participant sex, and anatomical sensor location, suggesting that peripheral temperature measurements are influenced by both physiological state and measurement context. With respect to temporal variation, afternoon sessions generally demonstrated improved agreement between ST and CBT compared with morning sessions. This was reflected in relatively smaller bias, narrower LoAs, and reduced variance of the CBT–ST difference across most anatomical sites and participant groups. While the magnitude of these differences was not uniform across all comparisons, the overall pattern suggests that peripheral temperature measurements may align more closely with internal thermal state later in the day under the conditions tested. This observation may reflect broader physiological or behavioral variation across time, although the present study was not designed to isolate specific underlying mechanisms.

A similar directional trend was observed when comparing activity conditions. Non-calm sessions generally exhibited more favorable agreement metrics than calm sessions, including lower variability and tighter LoA in several cases. This finding suggests that ST–CBT correspondence does not necessarily degrade under increased movement or activity, and in some cases may appear more stable during non-calm conditions. However, the extent of this effect varied across sites and participants, indicating that activity state interacts with other factors rather than acting as an independent determinant of agreement. Differences were also observed between male and female participants, although these should be interpreted cautiously given the sample size and exploratory nature of the study. In several conditions, females showed comparatively higher bias, wider LoA, and greater variability in ST–CBT differences, particularly during morning and calm sessions. Males, in contrast, tended to show lower variability and somewhat more stable agreement patterns in afternoon and non-calm conditions. These observations may reflect a combination of physiological and contextual factors, but the current dataset does not allow for definitive interpretation, and the findings should be regarded as preliminary. When evaluating the anatomical locations, the second-ranked site was considered the overall preferred skin site whenever the highest-ranked site corresponded to the forehead (Site 5). Although the forehead demonstrated favorable values, its use in firefighting applications is impractical because sensor placement at this location is incompatible with personal protective equipment (PPE), particularly due to interference from the helmet and flash hood. Consequently, the elbow and wrist sites were comparatively evaluated to identify the most suitable alternative sensor placement locations for practical implementation.

Across anatomical locations, consistent differences were observed in measurement performance. Elbow sites (Sites 2 and 4) generally demonstrated more favorable agreement with CBT than wrist sites (Sites 1 and 3), as indicated by lower bias, reduced LoA, and lower variability in ST–CBT differences. In addition, elbow sites showed slightly stronger correlations with CBT, although correlation strengths remained moderate overall. This suggests that while no single site provides a highly precise proxy for CBT under all conditions, some locations may offer relatively more stable measurement characteristics than others. Taken together, these results indicate that ST-based estimation of CBT is context-sensitive and influenced by both physiological state and sensor placement. In particular, elbow sites may offer a comparatively more reliable alternative to wrist sites when more direct measurement locations such as the forehead are not feasible. However, the observed variability across conditions highlights the need for cautious interpretation and further validation under operationally representative conditions, particularly in relation to wearable heat-risk monitoring applications.

Table 1 summarizes the variance of ST and CBT across all scenarios and measurement sites. This analysis was performed to characterize the distribution and consistency of the collected temperature dataset by examining within-scenario variability. The purpose of this analysis was to show the stability of temperature responses across measurement sites in all scenarios. To facilitate interpretation of ST variability, a heat map was developed using a red–green color scale, where green represents lower variance (indicating greater consistency) and red represents higher variance (indicating lower consistency) for each scenario. In comparison, CBT demonstrated minimal variation across scenarios and generally showed the lowest variance values in the male participant group (scenarios E-F).

Table 2 summarizes the variance of the CBT–ST difference for each scenario at each sensor site. A lower variance indicates less discrepancy between CBT and ST, reflecting stronger agreement between the two measurements. Priority was given to wrist and elbow sites in alignment with occupational and practical considerations for firefighters’ scenarios of fieldwork, with their PPE considering the helmet and flash hood positioned around the forehead area. Firefighters operate in highly dynamic environments where local heat exposure can vary depending on body orientation relative to the heat source, movement patterns, and task execution strategies [33]. Bilateral thermal symmetry is generally expected under resting and controlled conditions; however, this symmetry may not necessarily persist during physically demanding and asymmetric occupational activities [34]. Furthermore, personal protective equipment (PPE) does not always create identical thermal microenvironments on both sides of the body. Differences in sleeve or glove fit, donning tension, equipment positioning, and air-gap characteristics may influence local heat transfer and sweat evaporation at the wrist and elbow regions.

In six of the eight scenarios, the elbow sites (Sites 2 and 4) exhibited the lowest variance, suggesting that these sites generally provided the best correspondence with CBT. The second-ranked site was considered the overall best skin site whenever the highest-ranked site corresponded to the forehead (Site 5). However, in Scenarios E and F, representing the male morning sessions under calm and non-calm conditions, the wrist site (Site 3) demonstrated the lowest variance and therefore the strongest agreement with CBT.

**Figure 3:**
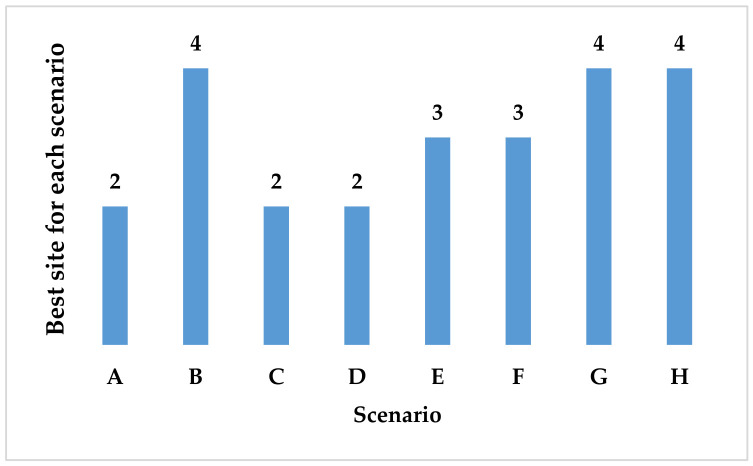
The graph of the best site for each scenario considering variance of (CBT-ST).

Figure 3 represents the graph of the best site for each scenario, which is presented in Table 2.

Table 3 summarizes the LoAs and bias values obtained from the Bland–Altman analysis performed in SPSS (Version 30) for each scenario and sensor site. Lower absolute bias and narrower LoA indicate better agreement between ST and CBT. Although the Bland–Altman bias and LoA values observed in this study were relatively large, the method was applied primarily to identify the sensor site that demonstrated the closest agreement with CBT, rather than to establish interchangeability between ST and CBT. Bland–Altman analysis was selected because it is widely used to compare new or alternative temperature measurement approaches against gold-standard CBT methods. However, it is important to note that agreement between two CBT measurement methods typically involves substantially smaller differences than those observed in the present study, whereas the current analysis compares CBT with ST, for which the raw temperature values differed by ~10 °C. Accordingly, the Bland–Altman analysis in Table 3 was used in this study as a comparative tool to rank sensor sites by relative agreement with CBT and to identify the most suitable skin location for monitoring, rather than to assess whether ST can directly replace CBT measurements. After obtaining LoA and bias values, the overall best site indication was selected based on the lowest LoA and bias values as presented in Table 4. The second-ranked site was considered the overall best skin site whenever the highest-ranked site corresponded to the forehead (Site 5). Across the eight scenarios, the elbow sites (Sites 2 and 4) consistently showed the strongest agreement with CBT. Based on the Bland–Altman analysis, the elbow sites exhibited the narrowest LoA in six of the eight scenarios and the lowest bias in all eight scenarios.

**Figure 4:**
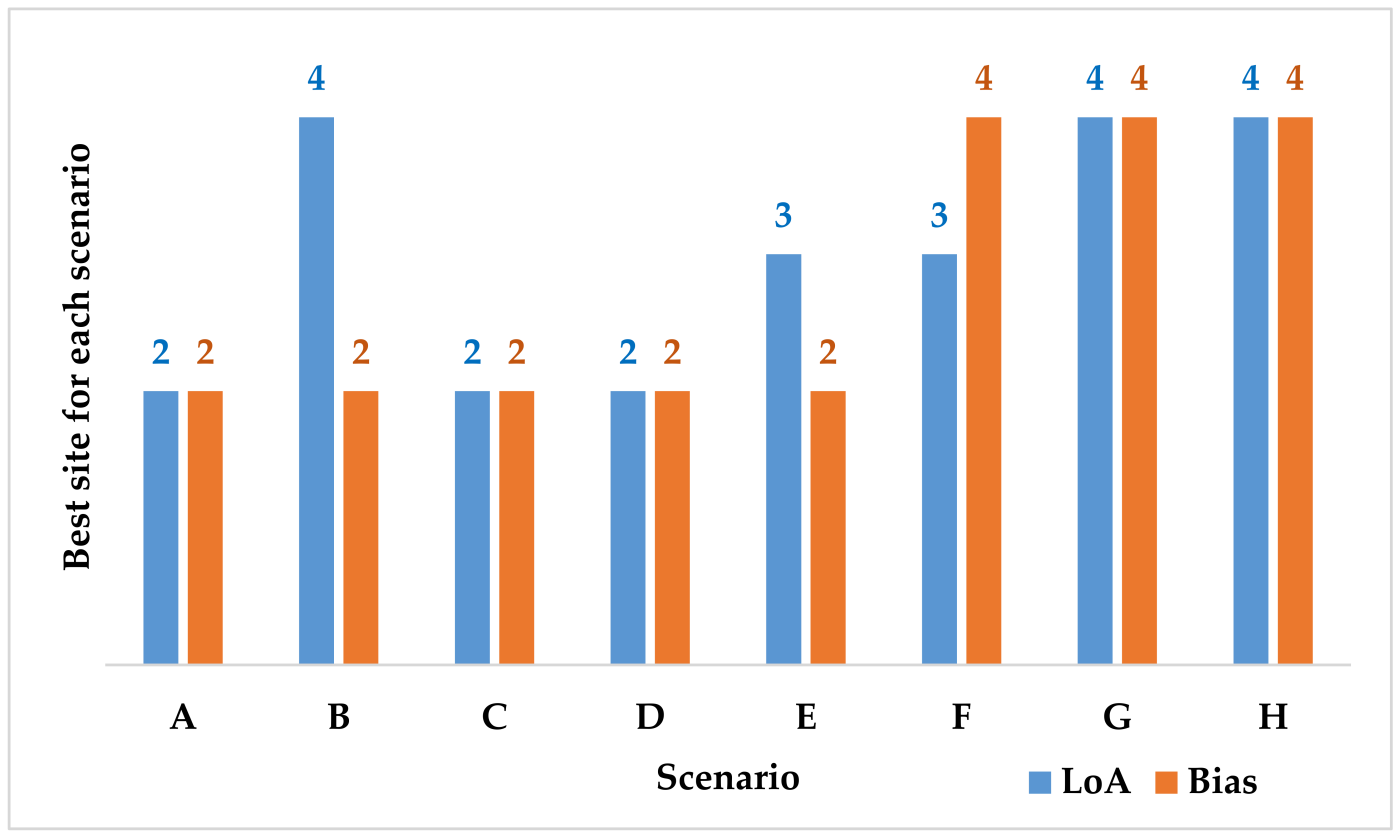
The graph of the best site for each scenario considering the Bland–Altman method.

Figure 4 represents the graph of the best site for each scenario considering the Bland–Altman method in Table 3.

The following subsections examine the effects of time of day, activity state, and sex on the relationship between ST and CBT. The results are presented to highlight how these factors influenced measurement variability and agreement across the different sensor sites. Together, these analyses provide further insight into the conditions under which specific skin sites may better approximate CBT.

Relative humidity remained relatively stable throughout the study period (3 days), with averages for each day ranging from 62.5% to 60.1%.

## 4. Discussion

This discussion examines the observed relationships between ST and CBT across temporal, behavioral, gender, and anatomical factors, and considers their implications for the design of non-invasive wearable heat-risk monitoring systems, particularly for sensor placement. The findings indicate that the relationship between ST and CBT is influenced by contextual and individual variability rather than remaining stable across conditions. Lower variability between ST and CBT during afternoon measurements suggests that time-of-day may affect the consistency of temperature signals and, consequently, the suitability of particular sensor placement. Similarly, reduced variability under non-calm conditions compared with calm states indicates that activity level may influence temperature signal behavior and sensor performance. Differences observed between male and female participants further highlight the importance of considering sex-related variability in wearable sensing design and interpretation. Across anatomical locations, elbow sites demonstrated closer agreement with CBT than wrist locations, suggesting a more promising direction for non-invasive and on-body sensor placement in wearable heat-monitoring applications.

The findings are interpreted from a wearable sensing systems perspective rather than treating ST as a direct physiological replacement for CBT. Within this context, measurement performance is understood as being shaped by interacting factors including sensor placement, activity state, time of day, and individual variability. Accordingly, the discussion focuses on how these factors may inform the design, deployment, and interpretation of temperature-based wearable sensing systems intended for non-invasive heat-risk monitoring.

### 4.1. Time-of-Day Effect: Afternoon Compared with Morning

The results indicate a consistent temporal pattern in which afternoon sessions demonstrated comparatively closer agreement between ST and CBT than morning sessions. Across sexes and anatomical sites, afternoon measurements were generally associated with smaller bias, narrower LoA, and lower variance in the differences between ST and CBT. Although the magnitude of these differences varied across sites and participant groups, the overall trend suggests that the relationship between peripheral and internal temperature measurements may be influenced by time-of-day effects. The most pronounced improvements in agreement were observed at the elbow sites, indicating that temporal influences may not affect all body locations uniformly.

While the present study was not designed to identify the underlying physiological mechanisms, the observed variation may plausibly relate to diurnal changes in thermoregulatory processes, peripheral perfusion, or skin blood flow, all of which may affect the extent to which peripheral temperature reflects internal thermal status [35,36]. Environmental or behavioral differences between sessions may also have contributed to the observed patterns. Consequently, these findings should not be interpreted as suggesting that afternoon measurements are inherently superior, but rather that time of day represents a potentially important source of variability in ST–CBT agreement. For wearable heat-monitoring systems, the results highlight the importance of considering temporal variability during sensor development and interpretation of temperature-based physiological measurements.

### 4.2. Activity-State Effect: Non-Calm Compared with Calm

The comparison between calm and non-calm sessions suggests that activity state may influence the relationship between ST and CBT. Overall, non-calm sessions were associated with smaller bias, narrower LoA, and lower variance in the differences between ST and CBT compared with calm sessions. Within the context of this study, these findings suggest that peripheral temperature measurements tended to correspond more closely with CBT during non-calm conditions. Although the observed improvements were modest in some cases and not entirely uniform across all sites or participants, the direction of the trend remained broadly consistent.

This pattern is notable because increased movement or reduced stillness did not appear to substantially degrade agreement, as may be expected if motion-related artefacts were the dominant influence on signal quality [36]. Instead, the findings suggest that activity-related physiological responses may contribute to more stable or representative peripheral thermal behavior under certain conditions. One possible explanation is that increased movement during non-calm conditions may improve blood circulation and skin blood flow, allowing skin temperature to reflect internal body temperature more consistently [36,37]. However, this interpretation remains speculative and cannot be confirmed from the present study alone. In contrast, during calm conditions, skin temperature may be more affected by short-term peripheral fluctuations or surrounding environmental conditions, particularly at exposed body locations. These interpretations should therefore be considered exploratory. Nonetheless, the findings indicate that behavioral state may represent an important contextual factor affecting the performance of peripheral temperature sensing. From a wearable systems perspective, this reinforces the need to evaluate temperature-based sensing approaches under realistic operational conditions rather than relying solely on resting or controlled-state measurements during system development and validation [15,21,38].

### 4.3. Sex Differences: Patterns of Variability Between Females and Males

Sex-related differences were observed in the agreement between ST and CBT, although these findings should be interpreted cautiously and within the limitations of the study context. Across several comparisons, female participants generally demonstrated larger bias, wider LoA, and greater variance in the differences between ST and CBT than male participants. These differences were most apparent during morning and calm sessions, where disagreement between peripheral and internal temperature measurements appeared comparatively greater among females. In contrast, male participants tended to exhibit lower variability, particularly during afternoon and non-calm conditions, where agreement metrics were often more favorable.

The findings suggest that the relationship between ST and CBT may not be entirely consistent across sexes and that variability in measurement agreement may be influenced by physiological or contextual differences between participants. However, the present study was not designed to determine the causes of these differences, and the results should not be interpreted as indicating a universal or intrinsic sex effect. Multiple factors may contribute to the observed patterns, including differences in peripheral circulation, body composition, thermoregulatory responses, hormonal influences, or broader between-subject variability, none of which were directly assessed in this work. In addition, the relatively limited sample size and subgroup distribution may have influenced the apparent magnitude of the observed differences. Accordingly, these findings are best understood as preliminary observations that warrant further investigation rather than definitive conclusions. Nevertheless, from a wearable sensing perspective, the results suggest that user-specific variability may influence model performance and that sex-related factors may merit consideration during development, validation, or calibration of temperature-based heat-monitoring systems.

### 4.4. Site Hierarchy: Elbow Sites Compared with Wrist Sites

Across the anatomical locations examined, elbow sites generally demonstrated more favorable agreement with CBT than wrist sites. Elbow measurements were typically associated with lower bias, narrower LoA, and lower variance in the differences between ST and CBT across multiple conditions. Correlations between ST and CBT also tended to be somewhat higher at the elbow locations, although these relationships remained modest overall and should not be interpreted as evidence of strong linear correspondence. Collectively, these findings suggest that elbow sites may provide comparatively more stable and informative peripheral temperature measurements than wrist locations under the conditions examined in this study.

The consistency of this pattern across several analyses supports the practical relevance of sensor placement in wearable heat-monitoring systems. Although the present study cannot determine the precise reasons for the observed site differences, the comparatively stronger performance of elbow locations may relate to reduced environmental exposure, differences in local tissue characteristics, or more stable thermal behavior relative to the wrist. In contrast, wrist sites may be more susceptible to ambient temperature influences, movement-related disturbances, or greater peripheral variability. These interpretations remain exploratory and would require targeted physiological investigation to confirm. Importantly, the findings do not suggest that elbow measurements can directly replace CBT measurement, but rather that elbow sites may offer relatively more favorable conditions for peripheral sensing compared with wrist locations. From an applied wearable design perspective, this distinction is novel and provides a new direction for sensor placement for heat risk monitoring applications. In situations where forehead placement is considered impractical or incompatible with firefighters’ PPE due to interference with the helmet and the flash-hood, the elbow may represent a better alternative location for temperature-based non-invasive heat risk monitoring, although further validation under operational firefighting conditions would be necessary before stronger placement recommendations can be made.

## 5. Limitation

The study was exploratory with a relatively limited sample size, which constrains generalizability. The participants in this study were not firefighters and were not wearing firefighting gear.

## 6. Conclusions

Overall, the results indicate that ST–CBT agreement is influenced by contextual and measurement-related factors such as activity state, time of the day, sex differences, and site hierarchy. Slightly improved agreement was observed in afternoon compared with morning sessions, reflected in reduced bias and narrower limits of agreement across several comparisons. A similar tendency was observed for activity state, where non-calm conditions showed comparable or marginally improved agreement relative to calm conditions. Differences across anatomical locations were also observed, with elbow sites generally demonstrating closer agreement with CBT than wrist sites, although the magnitude of this effect varied across conditions. In addition, sex-related variability in agreement metrics suggests that inter-individual differences may influence peripheral temperature behavior, although these findings remain exploratory. These results highlight that peripheral temperature sensing in wearable systems is strongly influenced by context and body location, rather than reflecting a stable representation of core thermal state. As such, they reinforce the importance of considering physiological variability and sensor–body interaction when designing wearable heat-monitoring systems intended for real-world deployment. In particular, the findings suggest that sensor placement decisions and interpretation of temperature signals should account for temporal, behavioral, and individual differences.

As explained in Section 3, while our study measures the relevant bilateral differences in participants during calm and non-calm states and did not measure actual asymmetric firefighting tasks, we believe this work presents a starting point for considering positionality of wearables in dynamic environments of use. Future work will focus on validating these findings in larger and more diverse populations, and with operational firefighter cohorts wearing firefighting PPE gear, considering handedness without assuming equivalence between left and right sides. Future studies must also consider ecologically representative conditions such as operational training activities. In those future studies, a critical challenge will be methodological consideration of data collection under the exposure of radiant heat during petrochemical and compartment fire behavior training in live-fire contexts.

## Figures and Tables

**Table 1:** Variance of ST and CBT for each scenario for each site.

Scenario	ST (°C^2^)					CBT (°C^2^)
	Site 1	Site 2	Site 3	Site 4	Site 5	
A	6.310	3.370	7.102	4.291	4.314	0.071
B	3.383	1.683	4.370	1.199	0.680	0.086
C	2.015	1.366	1.828	1.694	0.689	0.069
D	1.882	0.745	1.515	1.541	0.904	0.069
E	3.223	3.680	3.106	4.493	4.747	0.028
F	1.224	3.018	0.787	1.454	1.113	0.037
G	1.290	1.473	2.737	1.111	0.444	0.043
H	1.591	1.251	1.190	0.754	0.588	0.047

Green represents lower variance and red represents higher variance for each scenario.

**Table 2:** Variance of (CBT-ST) for each scenario.

Scenario	Site 1 (°C^2^)	Site 2 (°C^2^)	Site 3 (°C^2^)	Site 4 (°C^2^)	Site 5 (°C^2^)	Best Site	2nd Best Site	Overall Best Site Indication
A	6.55	3.60	7.26	4.56	4.18	2	5	2
B	4.02	2.14	4.64	1.51	0.75	5	4	4
C	2.17	1.67	1.92	2.07	0.68	5	2	2
D	1.92	0.86	1.47	1.57	0.75	5	2	2
E	3.40	3.62	3.22	4.44	4.71	3	2	3
F	1.18	2.87	0.62	1.20	1.01	3	5	3
G	1.23	1.32	2.66	0.98	0.45	5	4	4
H	1.76	1.25	1.37	0.65	0.57	5	4	4

**Table 3:** LoA and Bias for each scenario. Supplementary Material shows Bland-Altman plots.

Scenario	Site 1 (°C)		Site 2 (°C)		Site 3 (°C)		Site 4 (°C)		Site 5 (°C)	
	LoA	Bias	LoA	Bias	LoA	Bias	LoA	Bias	LoA	Bias
A	10.032	8.14	7.437	5.867	10.56	7.74	8.37	6.113	8.018	5.167
B	7.863	7.663	5.732	5.483	8.44	7.943	4.823	5.663	3.396	4.51
C	5.773	7.307	5.068	5.08	5.43	7.8	5.645	5.713	3.238	4.533
D	5.43	7.82	3.627	4.793	4.748	7.62	4.91	5.227	3.395	5.107
E	7.232	6.893	7.458	5.6	7.035	6.867	8.262	5.663	8.504	6.14
F	4.25	6.153	6.636	4.86	3.09	5.987	4.29	4.48	3.932	5.053
G	4.352	6.883	4.495	4.683	6.388	6.837	3.871	4.517	2.633	4.563
H	5.2	6.4	4.388	4.32	4.583	6.353	3.156	4.26	2.969	4.407

**Table 4:** Overall best site indication for each scenario based on LoA and bias.

Scenario	LoA (°C)		Bias (°C)		Site Indication				Overall Best Site Indication	
	Lowest	2nd Lowest	Lowest	2nd Lowest	Lowest LoA	2nd Lowest LoA	Bias	2nd Lowest Bias	LoA	Bias
A	7.44	8.02	5.87	5.87	2	5	5	2	2	2
B	4.82	4.82	5.48	5.48	5	4	5	2	4	2
C	5.07	5.07	5.08	5.08	5	2	5	2	2	2
D	3.63	3.63	4.79	5.11	5	2	2	5	2	2
E	7.04	7.23	5.6	5.66	3	1	2	4	3	2
F	3.09	3.93	4.48	4.86	3	5	4	2	3	4
G	3.87	3.87	4.52	4.56	5	4	4	5	4	4
H	3.16	3.16	4.26	4.32	5	4	4	2	4	4

## Data Availability

Individual data are anonymized and confidential as per this study’s Human Ethics Approval. Access to aggregated data may be considered upon reasonable request to the corresponding author.

## Supplementary Materials

The following supporting information can be downloaded at: https://www.mdpi.com/article/10.3390/s26154837/s1.

## Abbreviations

The following abbreviations are used in this manuscript:

CBT	Core Body Temperature
ST	Skin Temperature
WST	Wearable Sensing Technology
NFPA	National Fire Protection Association
PPE	Personal Protective Equipment
LoA	Limits of Agreement
