# Supplementary material for: Investigating Wearable Sensor Placement for Heat Risk Monitoring of Firefighters: A Skin-Ear Temperature Study
Source: Sensors (Basel). 2026 Jul 31;26(15):4837. doi: 10.3390/s26154837 (PMC13469982; doi:10.3390/s26154837)

Investigating wearable sensors placement for heat risk monitoring of firefighters: a skin-ear temperature study

Supplementary Material: Bland-Altman plots for all sites (1,2,3,4,5) for each scenario

Y and X axis of the plots represent the difference and mean of the variables. Here are the 2 equations;

Y-axis: $(CBT - ST)$

X-axis: $\frac{CBT+ST}{2}$

According to the study design, the 8 scenarios are as follows.

A - Morning – Female - Calm
B - Morning - Female – Non-Calm
C - Afternoon - Female - Calm
D - Afternoon - Female – Non-Calm
E - Morning - Male - Calm
F - Morning - Male – Non-Calm
G - Afternoon - Male - Calm
H - Afternoon - Male – Non-Calm

Site 1 (A)

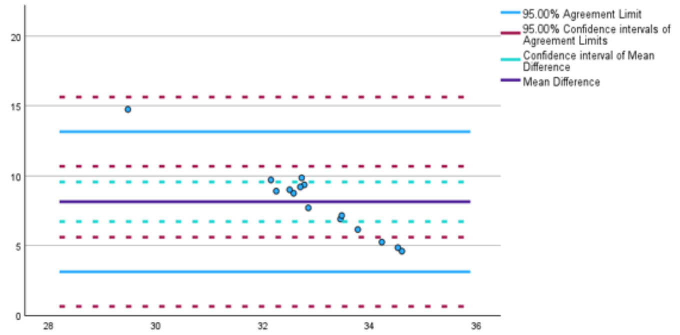

Site 1 (B)

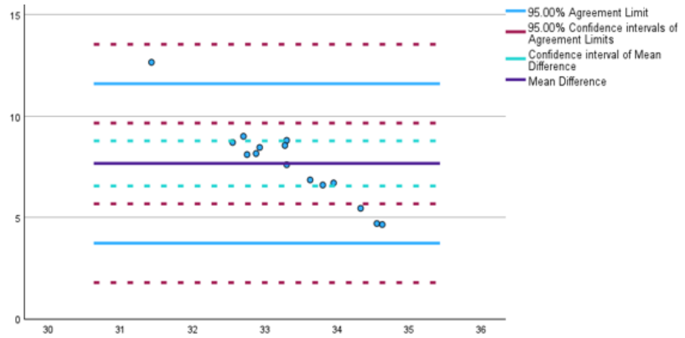

Site 1 (C)

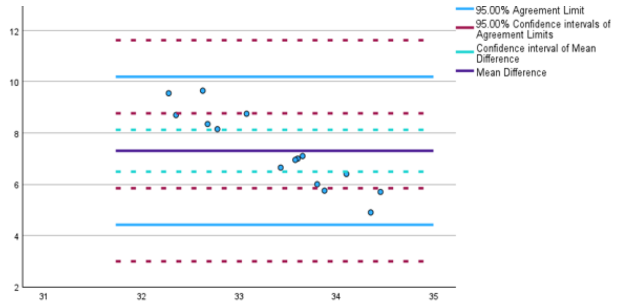

Site 1 (D)

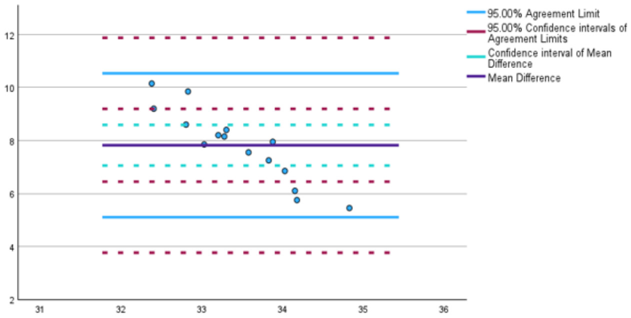

Site 1 (E)

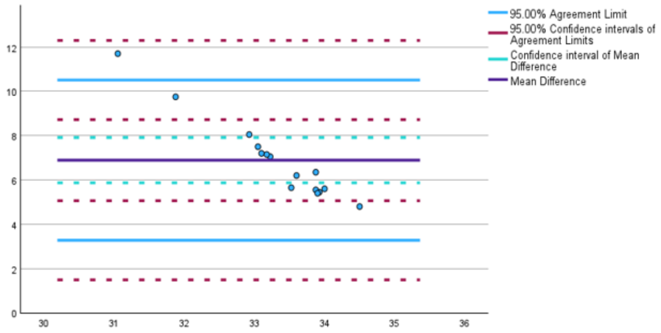

Site 1 (F)

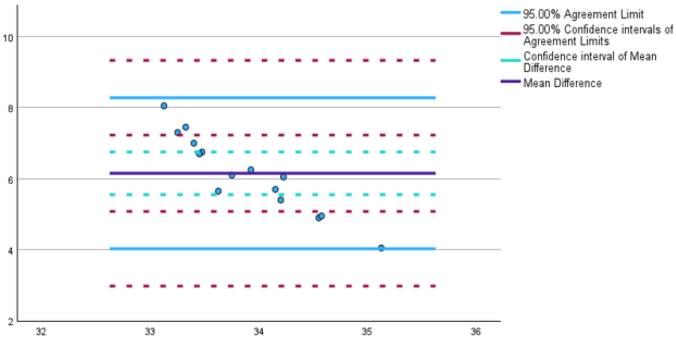

Site 1 (G)

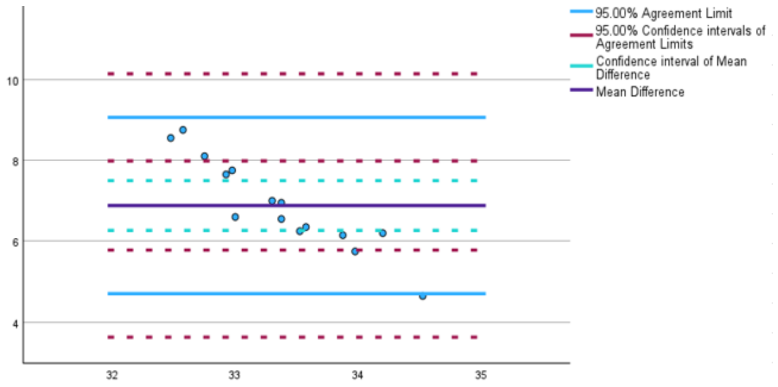

Site 1 (H)

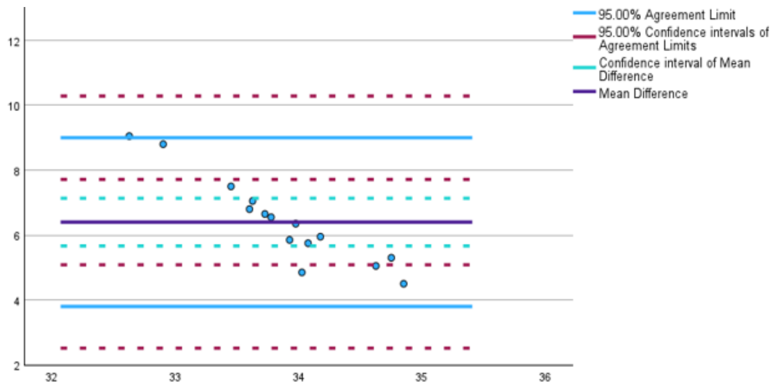

Site 2 (A)

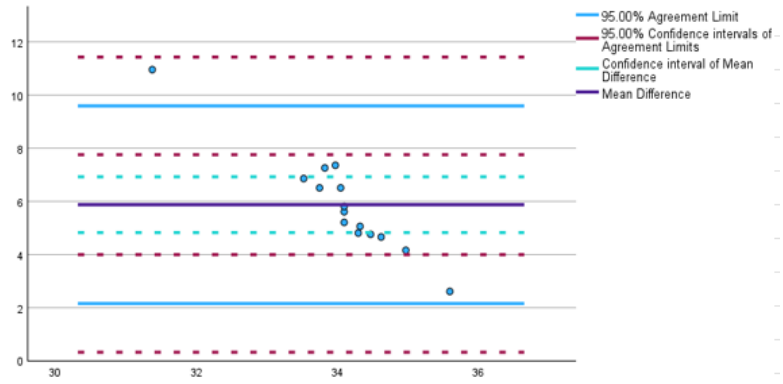

Site 2 (B)

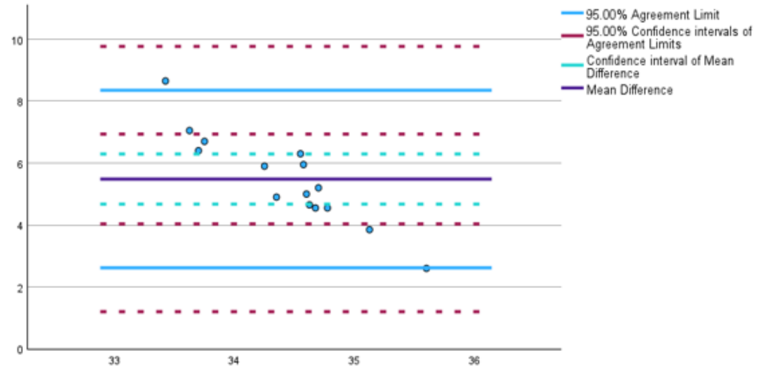

Site 2 (C)

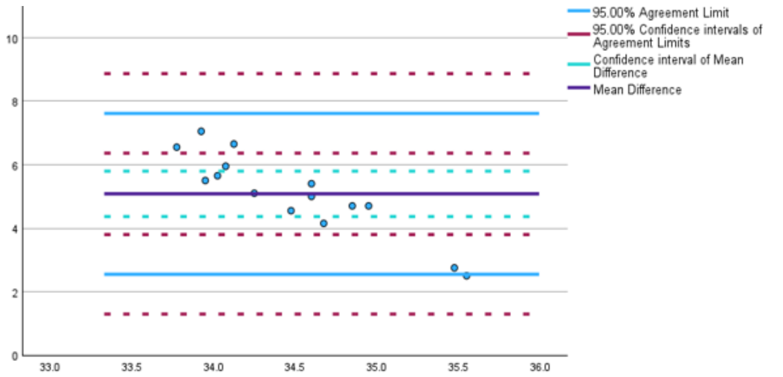

Site 2 (D)

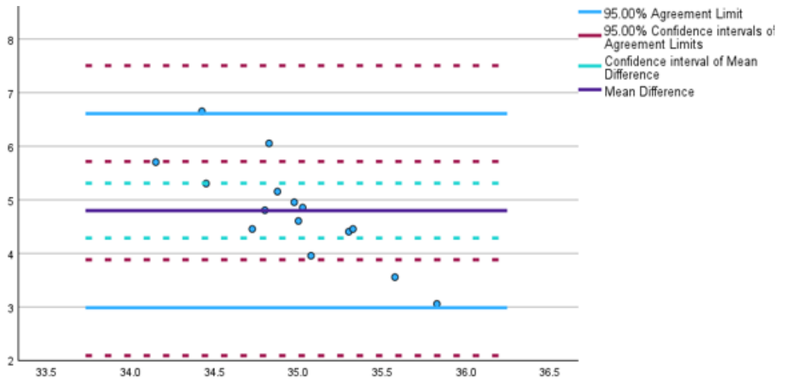

Site 2 (E)

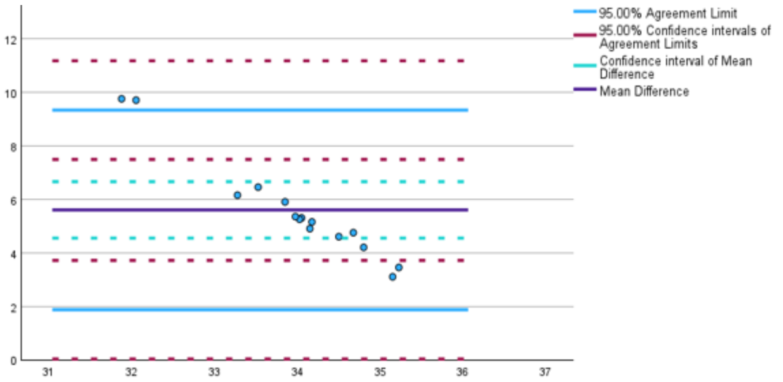

Site 2 (F)

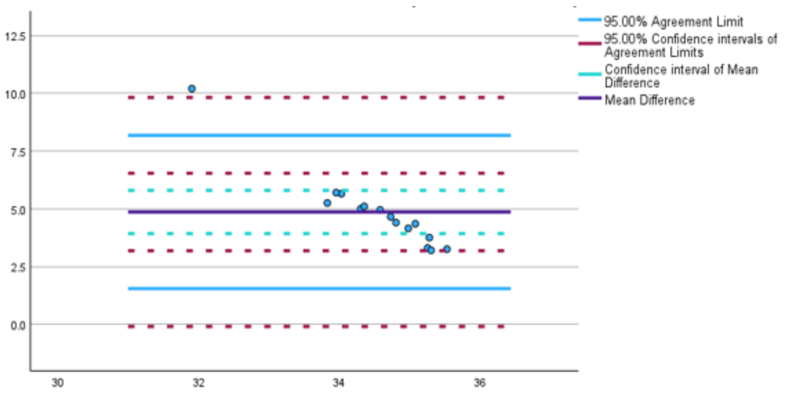

Site 2 (G)

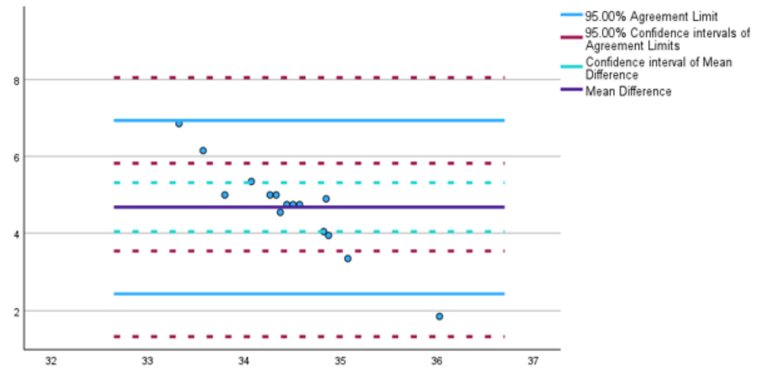

Site 2 (H)

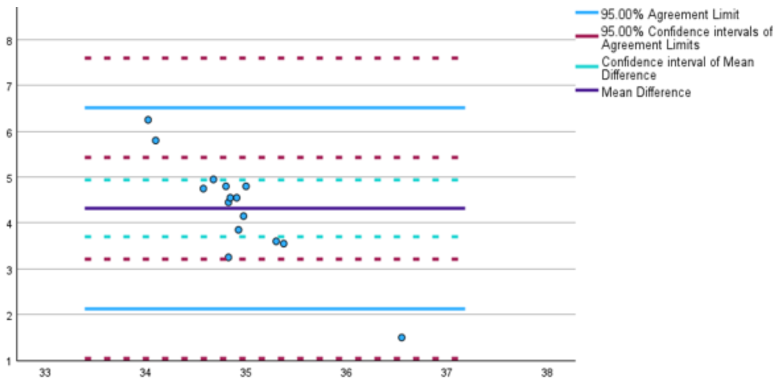

Site 3 (A)

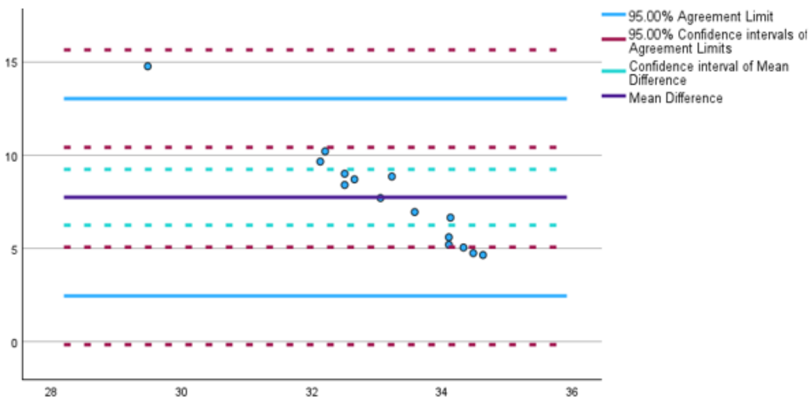

Site 3 (B)

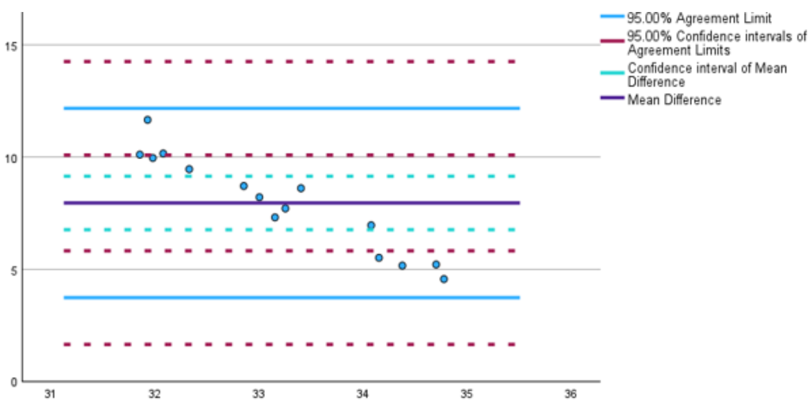

Site 3 (C)

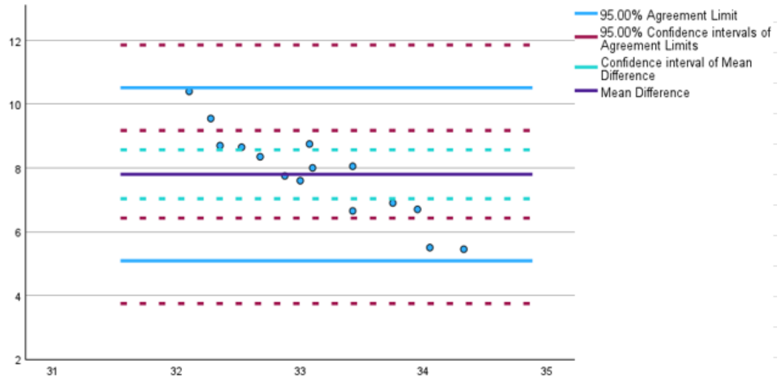

Site 3 (D)

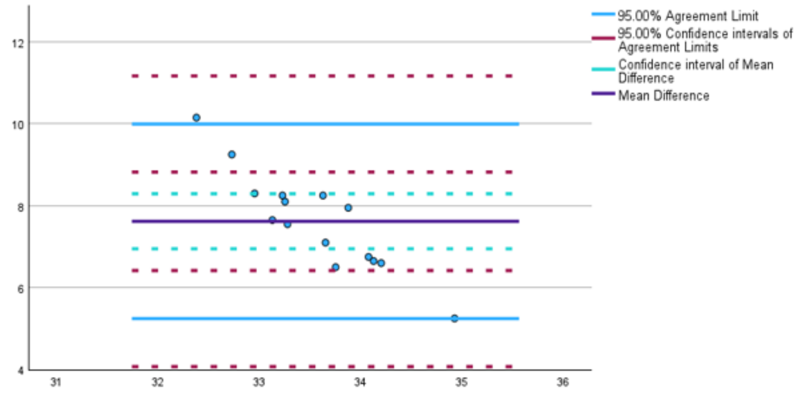

Site 3 (E)

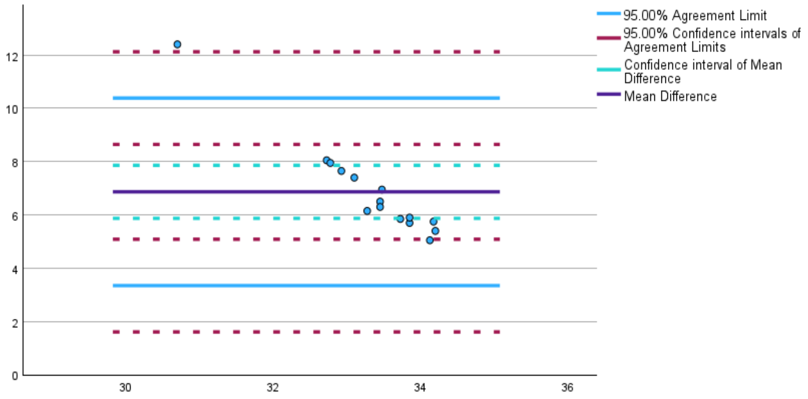

Site 3 (F)

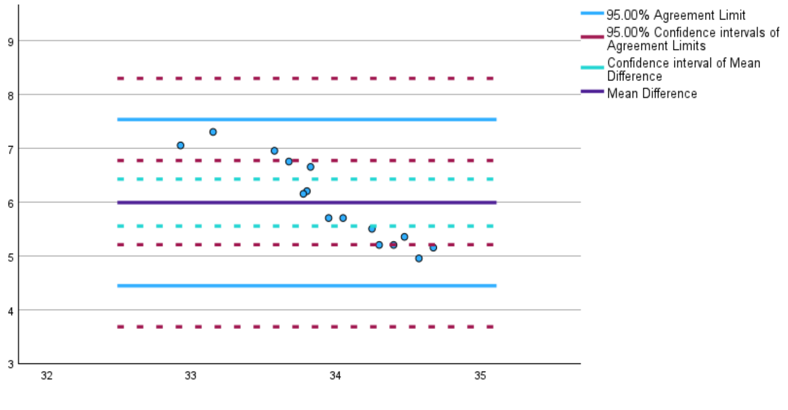

Site 3 (G)

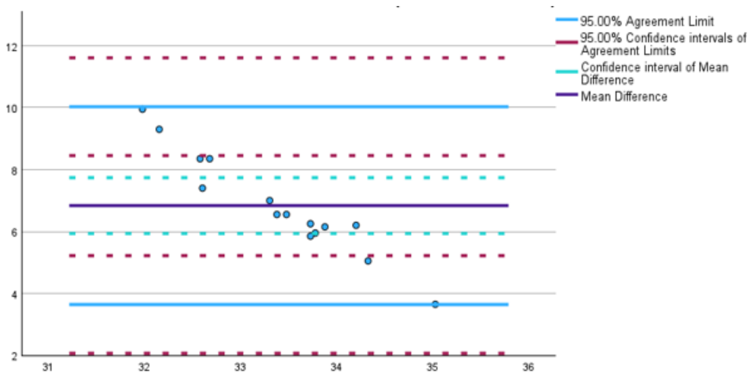

Site 3 (H)

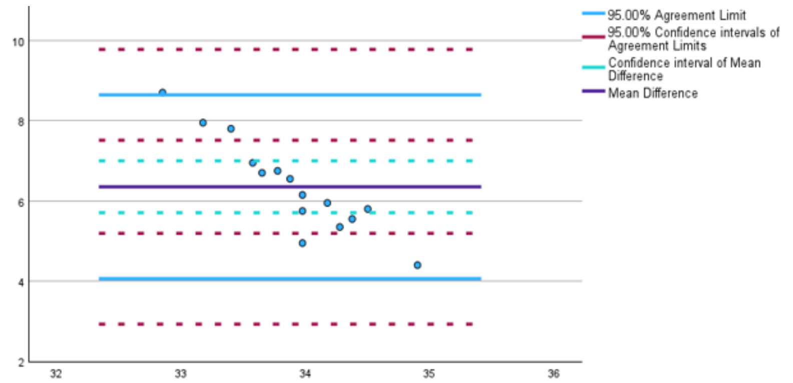

Site 4 (A)

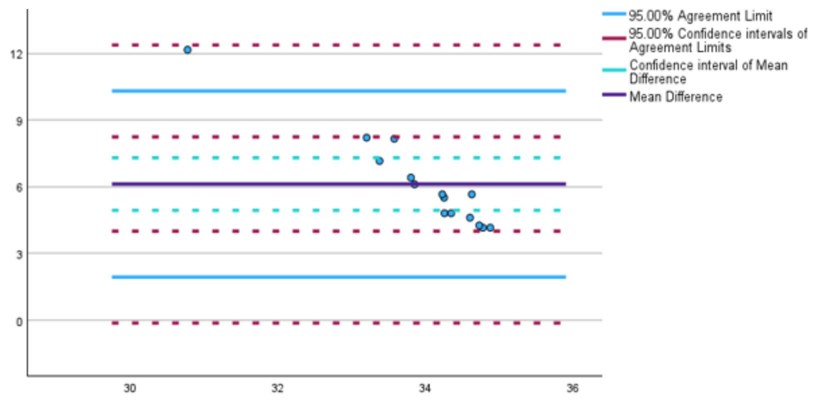

Site 4 (B)

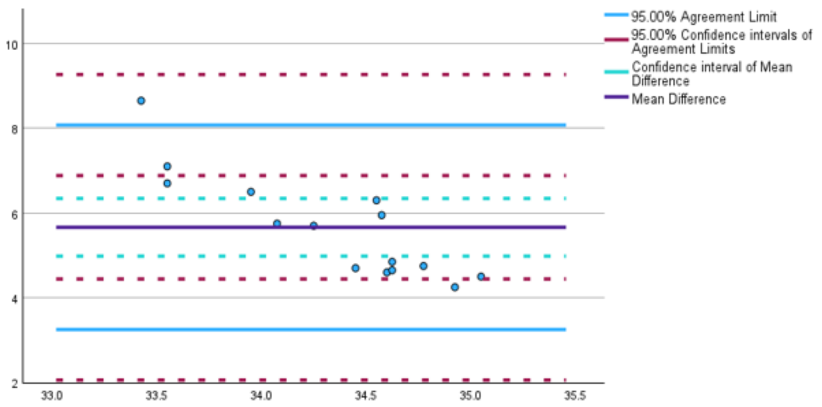

Site 4 (C)

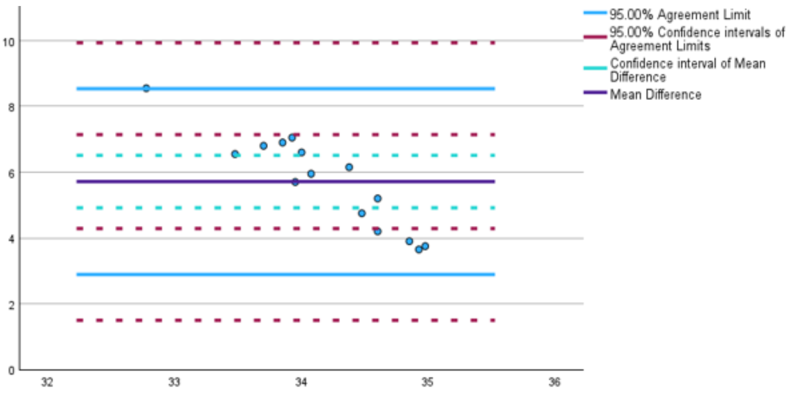

Site 4 (D)

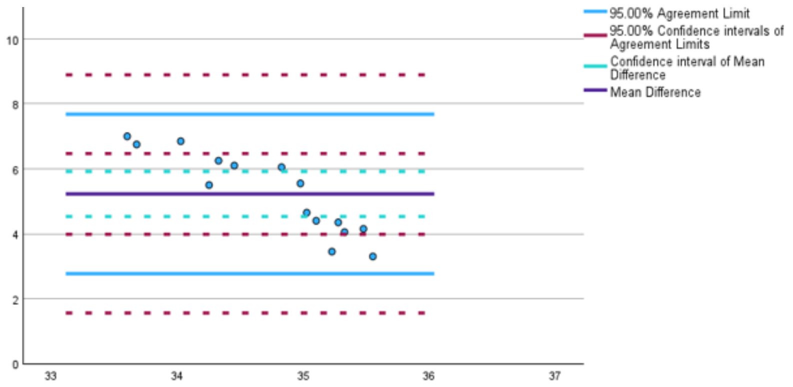

Site 4 (E)

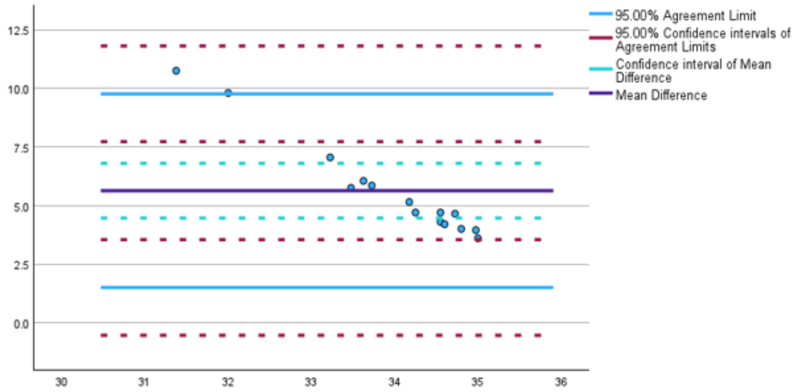

Site 4 (F)

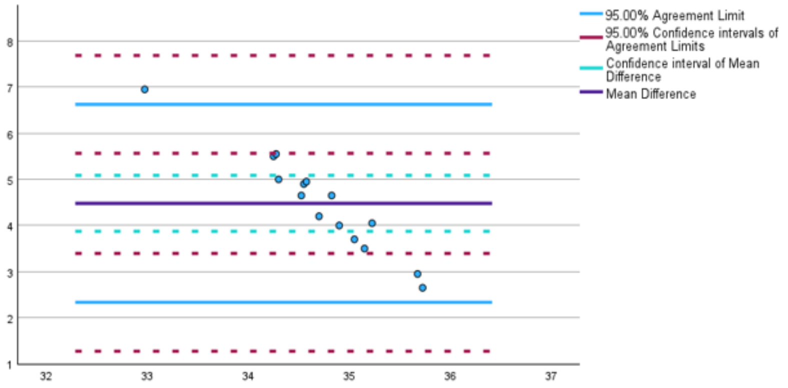

Site 4 (G)

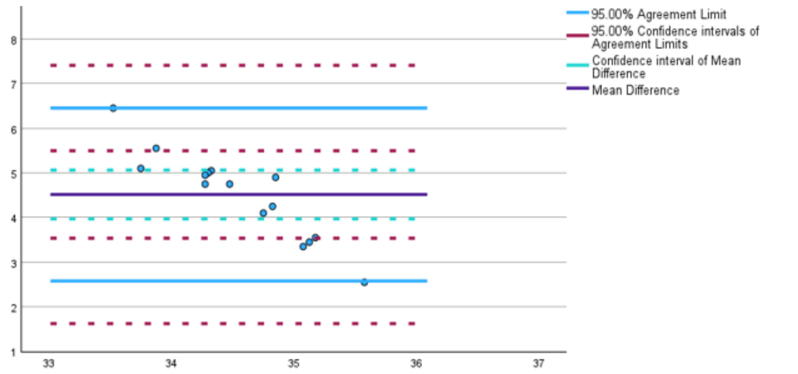

Site 4 (H)

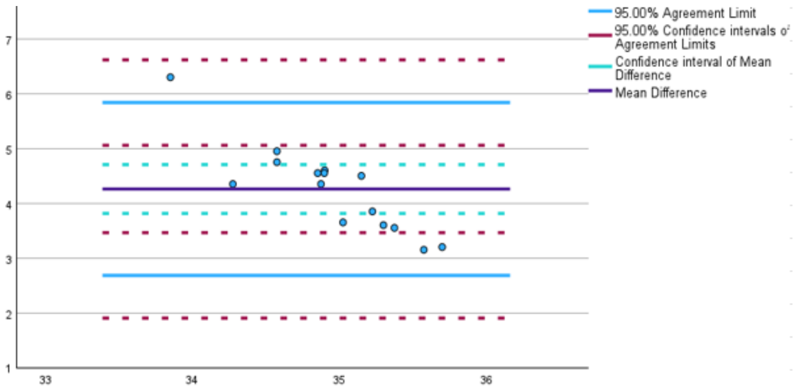

Site 5 (A)

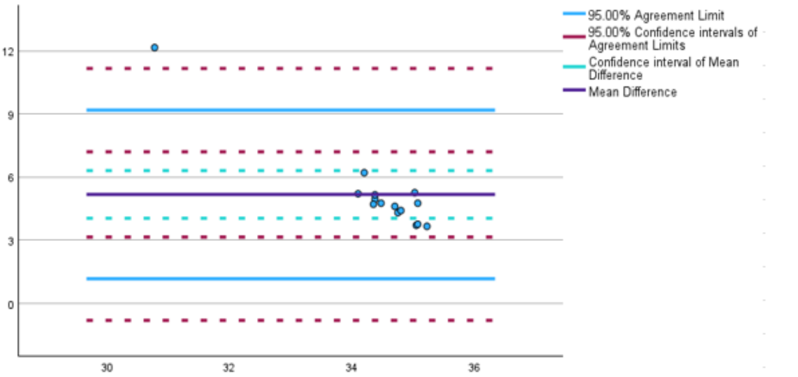

Site 5 (B)

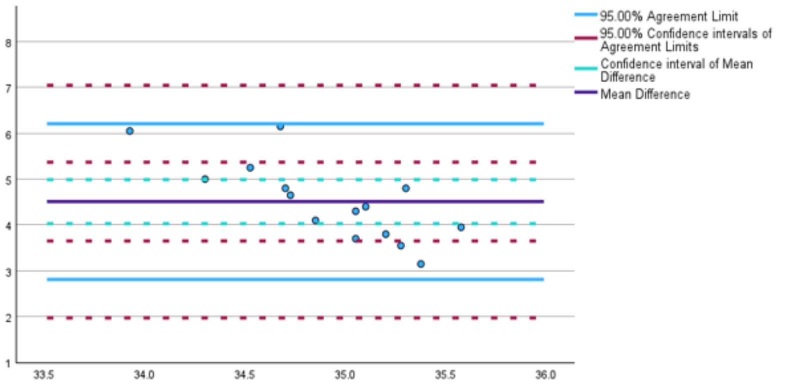

Site 5 (C)

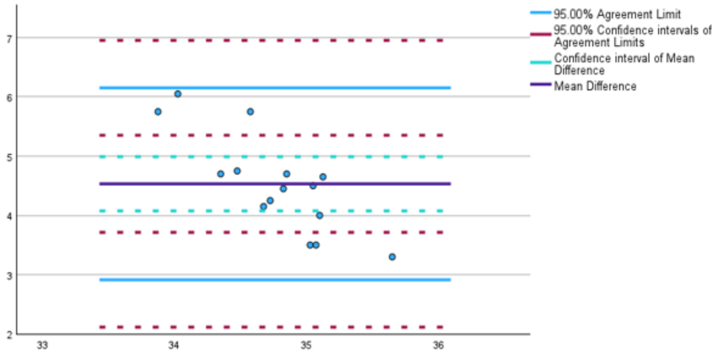

Site 5 (D)

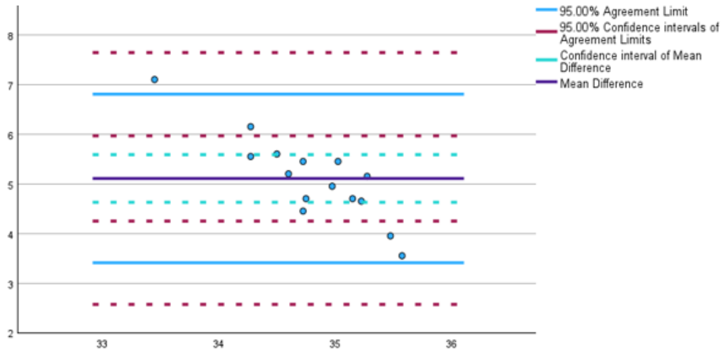

Site 5 (E)

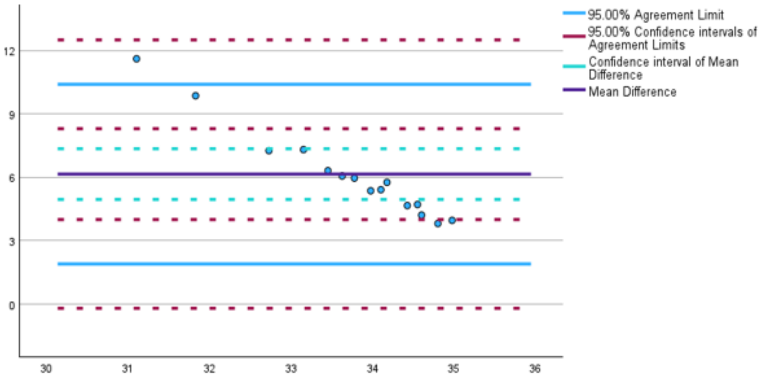

Site 5 (F)

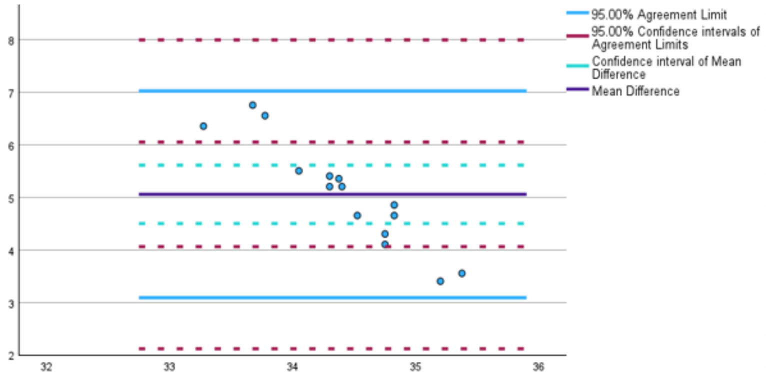

Site 5 (G)

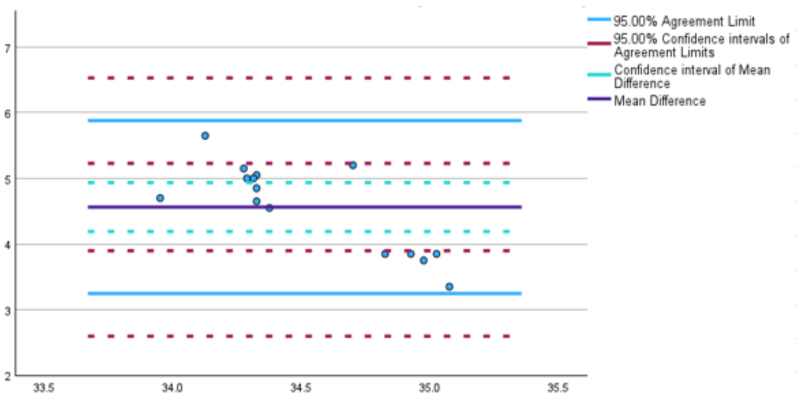

Site 5 (H)

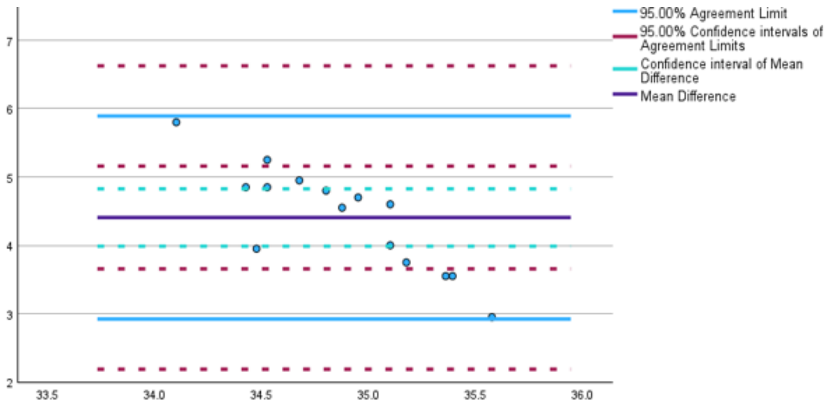

Supplement: Supplementary file 1 [file sensors-26-04837-s001.zip › sensors-4415308-supplementary.pdf]
